# *LEP* promoter methylation in the initiation and progression of clonal cytopenia of undetermined significance and myelodysplastic syndrome

**DOI:** 10.1186/s13148-023-01505-w

**Published:** 2023-05-26

**Authors:** Katja Kaastrup, Linn Gillberg, Stine U. Mikkelsen, Andreas D. Ørskov, Claudia Schöllkopf, Bo K. Mortensen, Bo Porse, Jakob W. Hansen, Kirsten Grønbæk

**Affiliations:** 1 The Epi-/Genome Lab, Department of Hematology, Rigshospitalet, Ole Maaløes Vej 5, 2200 Copenhagen, Denmark; 2 Department of Hematology, Rigshospitalet, Copenhagen, Denmark; 3 Biotech Research and Innovation Centre (BRIC), University of Copenhagen, Copenhagen, Denmark; 4 The Novo Nordisk Foundation for Stem Cell Research, Faculty of Health and Medical Sciences, University of Copenhagen, Copenhagen, Denmark; 5 Department of Hematology, Zealand University Hospital, Roskilde, Denmark; 6 Department of Hematology, Herlev Hospital, Herlev, Denmark; 7 The Finsen Laboratory, Rigshospitalet, Faculty of Health Sciences, University of Copenhagen, Copenhagen, Denmark

**Keywords:** MDS, CCUS, DNA methylation, Epigenetics, Leptin, Disease outcome, Prognosis, Leukemogenesis, Leukemic transformation, Biomarker

## Abstract

**Background:**

Idiopathic non-clonal cytopenia (ICUS) and clonal cytopenia (CCUS) are common in the elderly population. While these entities have similar clinical presentations with peripheral blood cytopenia and less than 10% bone marrow dysplasia, their malignant potential is different and the biological relationship between these disorders and myeloid neoplasms such as myelodysplastic syndrome (MDS) is not fully understood. Aberrant DNA methylation has previously been described to play a vital role in MDS and acute myeloid leukemia (AML) pathogenesis. In addition, obesity confers a poorer prognosis in MDS with inferior overall survival and a higher rate of AML transformation. In this study, we measured DNA methylation of the promoter for the obesity-regulated gene *LEP*, encoding leptin, in hematopoietic cells from ICUS, CCUS and MDS patients and healthy controls. We investigated whether *LEP* promoter methylation is an early event in the development of myeloid neoplasms and whether it is associated with clinical outcome.

**Results:**

We found that blood cells of patients with ICUS, CCUS and MDS all have a significantly hypermethylated *LEP* promoter compared to healthy controls and that *LEP* hypermethylation is associated with anemia, increased bone marrow blast percentage, and lower plasma leptin levels. MDS patients with a high *LEP* promoter methylation have a higher risk of progression, shorter progression-free survival, and inferior overall survival. Furthermore, *LEP* promoter methylation was an independent risk factor for the progression of MDS in a multivariate Cox regression analysis.

**Conclusion:**

In conclusion, hypermethylation of the *LEP* promoter is an early and frequent event in myeloid neoplasms and is associated with a worse prognosis.

**Supplementary Information:**

The online version contains supplementary material available at 10.1186/s13148-023-01505-w.

## Background

Myelodysplastic syndrome (MDS) comprises a heterogeneous group of hematopoietic stem cell disorders characterized by ineffective hematopoiesis, peripheral blood cytopenia, dysplasia, and increased risk of progression to acute myeloid leukemia (AML). MDS originates in early hematopoietic stem or progenitor cells (HSPC) and develops through genetic, epigenetic and immune aberrations of the HSPCs and their microenvironment [1, 2]. About 90% of MDS patients carry one or more oncogenic mutations [3-5], which frequently occur in genes that control DNA methylation (e.g., *TET2, DNMT3A* and *IDH1/2*). Indeed, abnormal promoter methylation has been shown to contribute to MDS pathogenesis and the number of differentially methylated CpG sites increases during disease progression [6, 7].

We and others have shown that myeloid malignancies are preceded by a long preclinical phase, where somatic mutations in myeloid cancer-associated genes accumulate in the HSPCs [8-12]. Patients with accompanying unexplained cytopenia, whose bone marrow does not fulfill the morphological criteria for MDS, are classified as having clonal cytopenia of undetermined significance (CCUS) [13]. Many of these patients develop MDS or AML over 1–10 years [10-12]. Unexplained cytopenia with neither somatic mutations nor morphological features of MDS are referred to as idiopathic cytopenia of undetermined significance (ICUS).

Whereas ICUS is generally an indolent disease [3], the clinical courses of CCUS and MDS are very heterogeneous ranging from indolent disease with minimal impact on survival to a smoldering disease with potential fast progression to high-risk MDS or AML [14, 15]. The molecular mechanisms of progression of CCUS are likely multifaceted, but we have recently shown that *TET2*-mutated CCUS is associated with hypermethylation of myeloid enhancers that are also methylated in AML, indicating that methylation may play a role [16].

A number of studies have shown an increased risk of cancer including MDS and AML in overweight and obese individuals [17-26]. Obesity conferred poorer prognosis in MDS patients with inferior overall survival (OS) and a higher rate of AML transformation [27]. The obesity-regulated gene *LEP* encodes the hormone leptin that besides regulating appetite and body mass plays a role in proinflammatory immune response, angiogenesis and lipolysis [28]. Recently, the *LEP* promoter was reported to be hypermethylated in MDS [29] and AML [30] patients. In AML patients, *LEP* hypermethylation was reported as an independent risk factor for shorter OS [30]. However, in MDS patients, *LEP* hypermethylation was associated with longer OS but was not an independent prognostic marker [29].

To investigate if *LEP* promoter hypermethylation is an early event in myeloid neoplasm, occurring also at a cytopenic state before dysplasia is present, and if it is associated with disease prognosis, we investigated *LEP* promoter methylation in patients with ICUS, CCUS and MDS and in longitudinal samples and evaluated its association with clinical parameters and disease outcome.

## Results

**Fig. 1:**
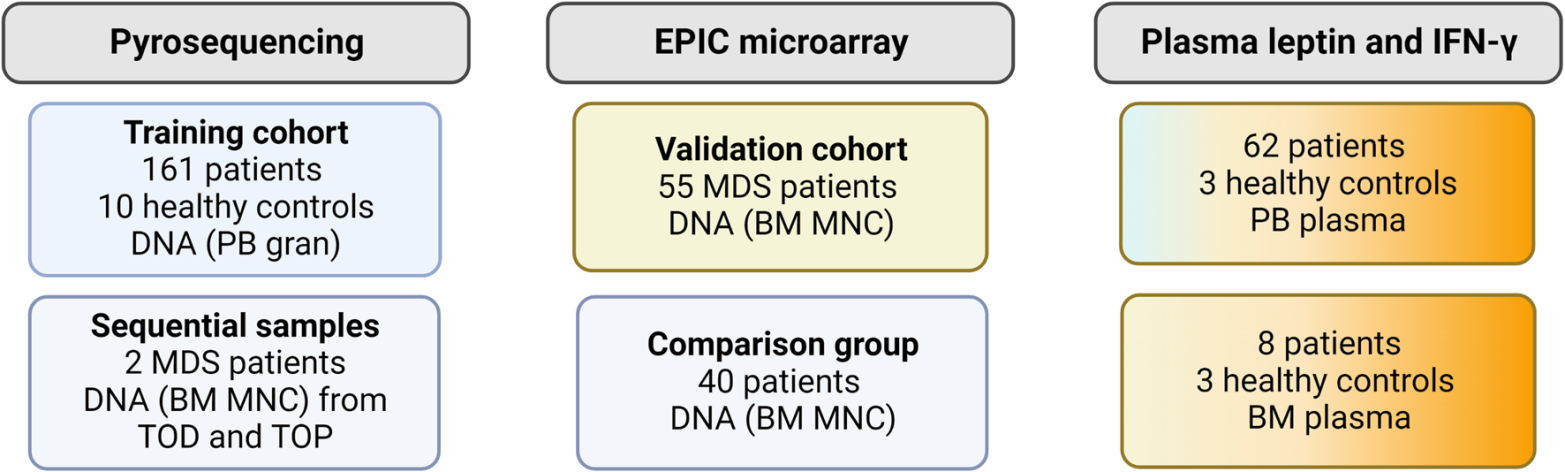
Overview of sample material included in each experiment. The color of the boxes represents material that are coming from patients included in several groups: blue boxes; patients were in the training cohort, yellow box; patients were part of the validation cohort: blue-orange boxes: 46 PB plasma samples are coming from patients who also have *LEP* promoter methylation data available measured with EPIC array. IFN-*γ*: interferon-*γ*; PB: peripheral blood; gran: granulocytes; BM: bone marrow; MDS: myelodysplastic syndrome; MNC: mononuclear cells; TOD: time of diagnosis; TOP: time of progression

The training cohort consisted of 65 ICUS patients, 39 CCUS patients, 57 MDS patients and 10 healthy controls (Fig. 1). The median age of diagnosis was 66, 70 and 74 years for patients with ICUS, CCUS and MDS, respectively (*p* < 0.001, Table 1). Patients with MDS had lower hemoglobin levels and neutrophil counts compared to patients with ICUS and CCUS (*p* < 0.001 and *p* = 0.007, respectively, Table 1). Of the MDS patients, 23% were high-risk patients (IPSS-*R* > 4.5, Table 1). Clinically reported mutations in MDS-related genes in the patients with CCUS and MDS are listed in Additional file 1: Table S1.

**Table 1:** Clinical characteristics of the training cohort

Characteristic	Controls, *N* = 10^a^	ICUS, * N* = 65^a^	CCUS, * N* = 39^a^	MDS, * N* = 57^a^	*p* value^b^
Age	59.0 (24.0, 66.0)	66.0 (17.0, 89.0)	70.0 (50.0, 89.0)	74.0 (26.0, 96.0)	** < 0.001**
Sex					0.272
Female	6 (60%)	23 (35%)	13 (33%)	16 (28%)	
Male	4 (40%)	42 (65%)	26 (67%)	41 (72%)	
Hgb		8.0 (4.5, 10.4)	6.7 (4.5, 8.7)	6.2 (4.8, 14.0)	** < 0.001**
Missing		0	0	9	
ANC		2.1 (0.3, 7.8)	2.0 (0.6, 8.0)	1.4 (0.3, 11.4)	**0.007**
Missing		2	0	11	
Platelets		126.0 (22.0, 417.0)	113.0 (18.0, 427.0)	115.0 (6.0, 666.0)	0.477
Missing		1	0	8	
IPSS-R category					> 0.999
Very low				3 (6.4%)	
Low				23 (49%)	
Intermediate				10 (21%)	
High				7 (15%)	
Very high				4 (8.5%)	
Missing				10	
No. of mutations					
0		54 (100%)	2 (5.1%)	3 (15%)	
1		0 (0%)	18 (46%)	6 (30%)	
2		0 (0%)	15 (38%)	5 (25%)	
3		0 (0%)	2 (5.1%)	5 (25%)	
4		0 (0%)	2 (5.1%)	1 (5.0%)	
Missing		11	0	37	

^a^Median (Range);* n* (%)

^b^Kruskal–Wallis rank-sum test; Fisher's exact test

*Hgb* hemoglobin, *ANC* absolute neutrophil count

Bold values denote statistical significance (*p* ≤ 0.05)

### ICUS, CCUS and MDS are associated with a hypermethylated *LEP* promoter

**Fig. 2:**
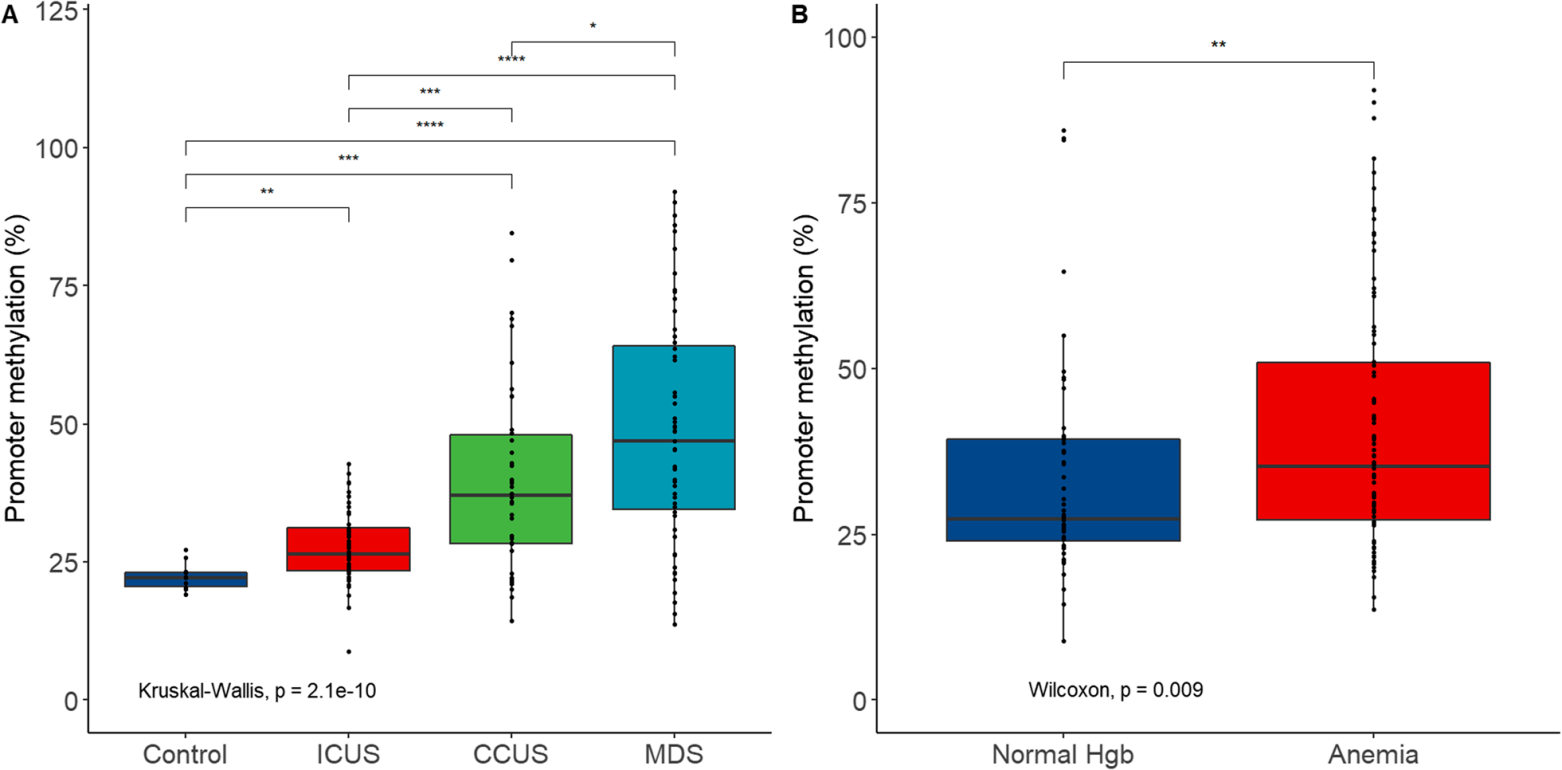
*LEP* promoter methylation in the training cohort in **A** patients with ICUS, CCUS or MDS and healthy controls and **B** patients with anemia or normal hemoglobin (Hgb). ICUS: idiopathic cytopenia of undetermined significance; CCUS: clonal cytopenia of undetermined significance; MDS: myelodysplastic syndrome; * indicate *p* ≤ 0.05; **indicate *p* ≤ 0.01; ***indicate *p* ≤ 0.001; ****indicate *p* ≤ 0.0001

We first investigated *LEP* promoter methylation using pyrosequencing in peripheral blood granulocytes from the training cohort. The average *LEP* promoter methylation was significantly higher in ICUS, CCUS and MDS patients compared to healthy controls (*p* = 2.1 × 10^–10^, Fig. 2A). In addition, *LEP* promoter methylation was significantly higher in CCUS and MDS compared to ICUS patients (*p* ≤ 0.001). Defining hypermethylation as mean DNA methylation above the median methylation level of the healthy controls plus two standard deviations (SD) (21.6 + 2.7%) revealed that 62% of ICUS, 77% of CCUS and 82% of MDS cases had a hypermethylated *LEP* promoter. Importantly, *LEP* promoter methylation was not significantly influenced by sex or age in a multivariate regression analysis (Additional file 1: Table S2).

Patients with anemia had higher *LEP* promoter methylation compared to patients with normal hemoglobin levels (*p* = 0.009, Fig. 2B). However, hemoglobin level was not significantly associated with *LEP* promoter methylation in a multivariate regression analysis including diagnosis, age, sex, neutrophil, and platelet count (Additional file 1: Table S2). There was no difference in *LEP* promoter methylation in patients with thrombocytopenia or neutropenia compared to patients with normal platelet and neutrophil counts, respectively (Additional file 2: Fig. S1).

### Hypermethylated *LEP* promoter is associated with a poor prognosis

When examining the methylation levels within each patient group, we observed large variations especially in CCUS and MDS patients with mean methylation levels ranging from 16 to 84% in CCUS and 13–93% in MDS patients (Fig. 2A). This prompted us to evaluate whether the clinical course of patients with a high *LEP* promoter methylation at the time of diagnosis was different compared to those with a lower *LEP* promoter methylation.

The median follow-up time was 22 months with an interquartile range of 16–32 months. The clinical outcome was inferior for patients with CCUS and MDS compared to patients with ICUS (Additional file 2: Fig. S2). MDS patients with a progressive disease during follow-up had a hypermethylated *LEP* promoter already at the time of diagnosis compared to those with a stable disease (*p* = 0.045), whereas this was not significantly different in CCUS patients (Additional file 2: Fig. S3).

**Fig. 3:**
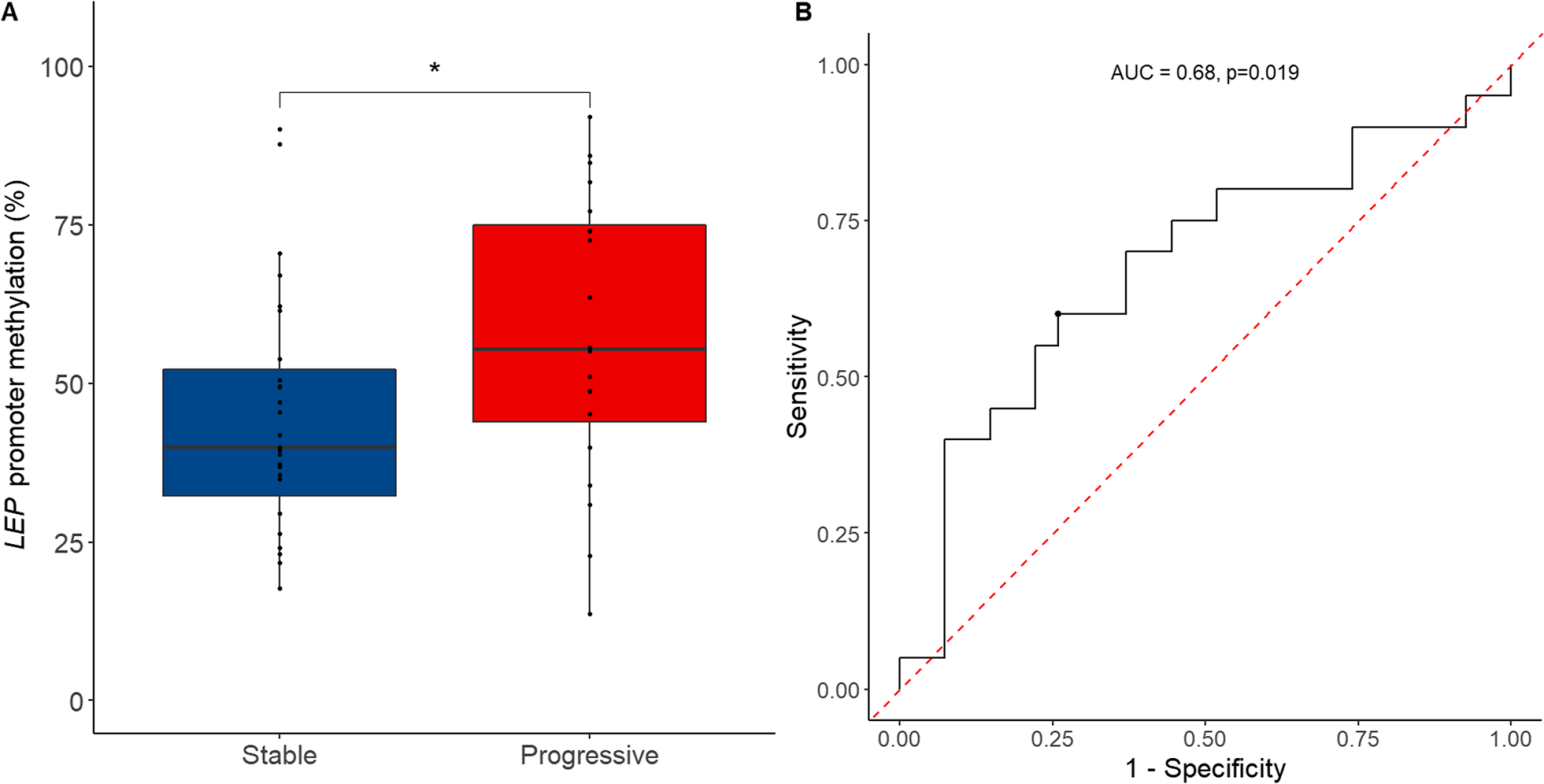
Prediction of outcome in MDS patients from the training cohort. **A** *LEP* promoter methylation at the time of diagnosis in MDS patients who during follow-up remain stable or progress. **B** ROC curve analysis of *LEP* promoter methylation separating stable and progressive MDS cases during follow-up. ROC: receiver operating characteristic curve; AUC: area under the curve; MDS: myelodysplastic syndrome; *indicate *p* ≤ 0.05

**Fig. 4:**
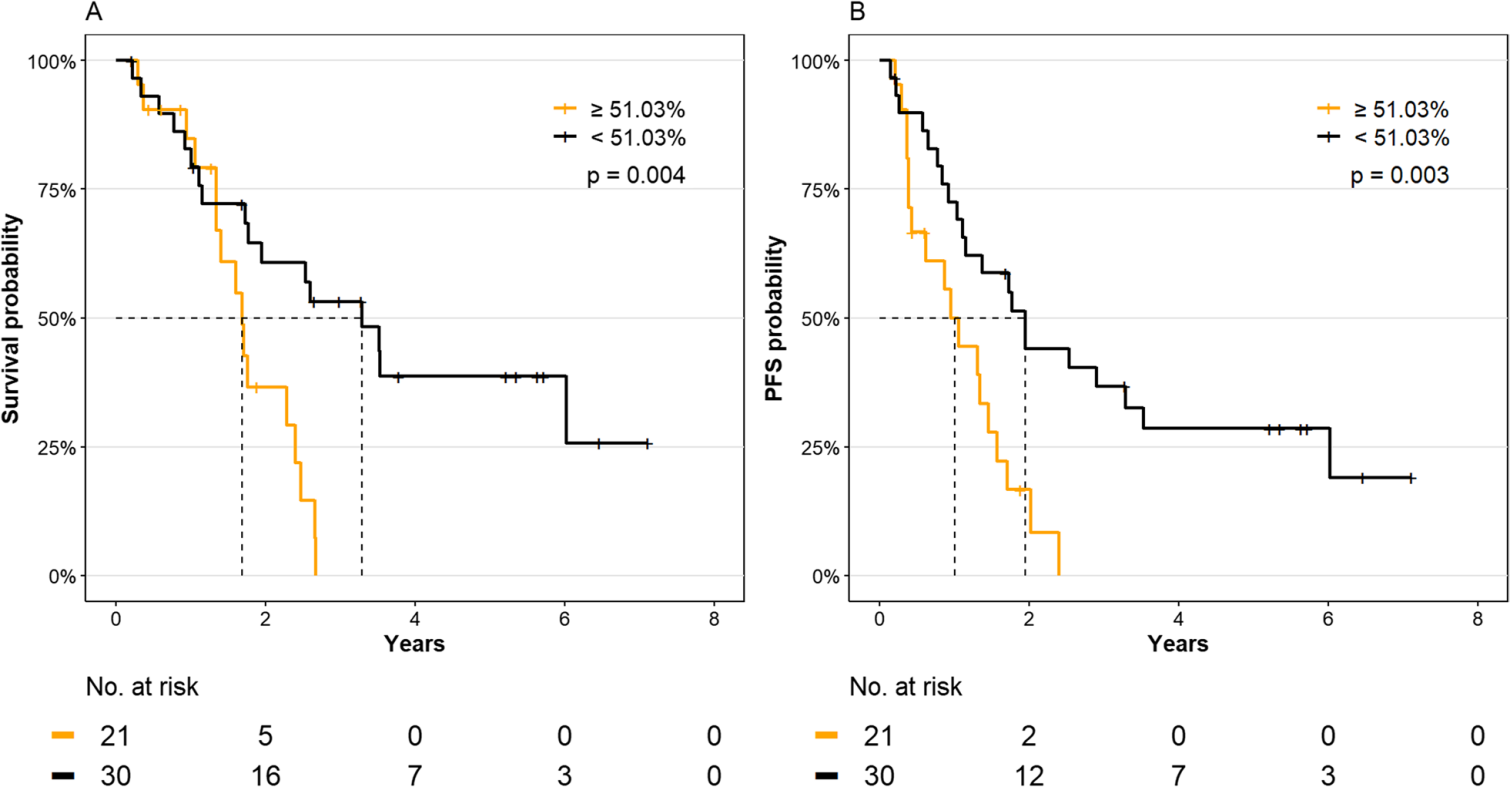
Impact of *LEP* promoter methylation in MDS patients on **A** overall survival, **B** progression-free survival (PFS). MDS: myelodysplastic syndrome

**Fig. 5:**
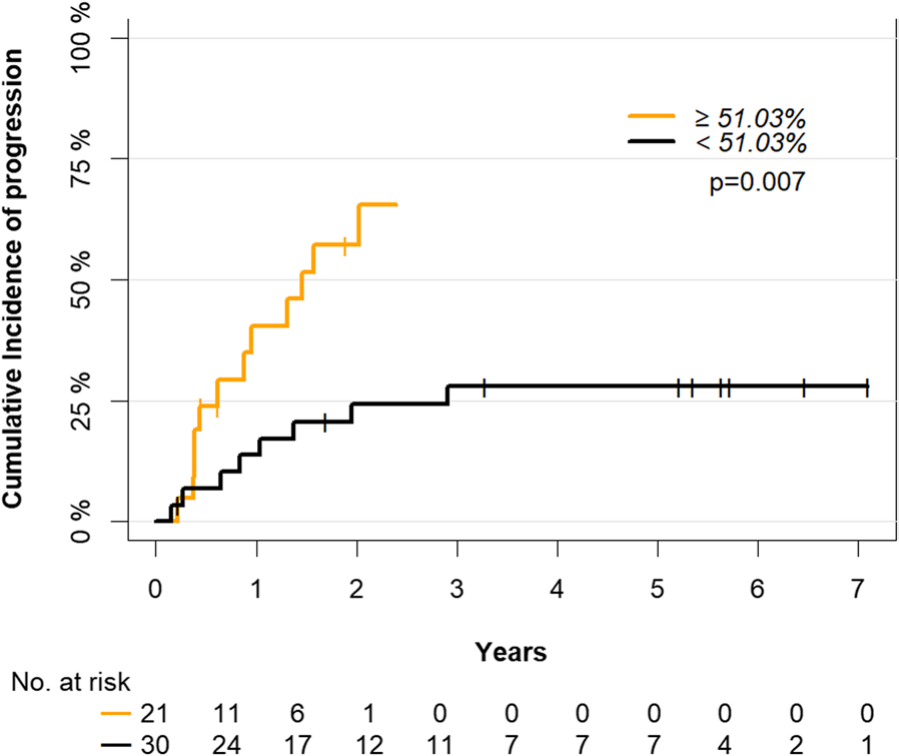
Prognostic value of *LEP* promoter methylation on cumulative risk of progression in MDS patients from the training cohort. MDS: myelodysplastic syndrome

A mean *LEP* promoter methylation above 51.03% at the time of diagnosis was able to stratify MDS patients with stable disease from patients with a progressive disease with a sensitivity of 60% and a specificity of 74% (positive predictive value = 63%, negative predictive value = 71%, and area under the curve (AUC) = 0.68, *p* = 0.019, Fig. 3B). Using this cutoff, we analyzed the effect of *LEP* promoter methylation on the cumulative incidence of progression and OS in MDS patients. Kaplan–Meier analysis indicated that MDS patients with a *LEP* promoter methylation above the cutoff had inferior OS (*p* = 0.004) (Fig. 4A). Similarly, MDS patients with a *LEP* promoter methylation above the cutoff had a significantly higher risk of progression (*p* = 0.007) (Fig. 5) and a shorter progression-free survival (PFS) (median PFS; 12 vs. 23.5 months, *p* = 0.003) (Fig. 4B). In a univariate Cox regression analysis, mean *LEP* promoter methylation was significantly associated with both progression and OS (Additional file 1: Table S3) and in a multivariate Cox regression analysis, mean *LEP* promoter methylation could serve as an independent marker for progression in MDS patients (HR = 4.36, 95% CI = 1.03–18.4, *p* = 0.045) (Table 2).

**Table 2:** Multivariate cox analyses of disease outcome in MDS patients from the training cohort

Characteristic	Time to death			Time to progression		
	HR	95% CI	*p* value	HR	95% CI	*p* value
*LEP promoter methylation*						
Below cutoff	–	–		–	–	
Above cutoff	1.92	0.69, 5.38	0.214	4.36	1.03, 18.4	**0.045**
*Sex*						
Female	–	–		–	–	
Male	0.81	0.18, 3.59	0.782	3	0.53, 17.0	0.216
Age	1	0.96, 1.04	0.87	1	0.95, 1.06	0.921
Hgb	0.58	0.40, 0.85	**0.005**	1.2	0.87, 1.67	0.271
ANC	1.08	0.87, 1.33	0.497	1.22	0.97, 1.53	0.089
Platelets	1	0.99, 1.00	0.378	1	0.99, 1.00	0.58
*IPSS-R*						
Low	–	–		–	–	
Intermediate	1.51	0.40, 5.76	0.548	1.83	0.41, 8.27	0.432
High	2.32	0.45, 11.8	0.312	1.97	0.23, 17.0	0.539
BM blast %	1.18	0.99, 1.40	0.067	1.11	0.94, 1.30	0.211

*HR* Hazard ratio, *CI* Confidence interval, *Below cutoff* mean *LEP* promoter methylation < 51.03%, *Above cutoff* mean *LEP* promoter methylation ≥ 51.03%, *Hgb* hemoglobin, *ANC* absolute neutrophil count, *IPSS-R* Revised International Prognostic Scoring System, *BM* bone marrow, *MDS* myelodysplastic syndrome

Bold values denote statistical significance (*p* ≤ 0.05)

In line with this, bone marrow blast percentages were significantly higher in the subgroup of MDS patients with a mean *LEP* promoter methylation above the cutoff (*p* = 0.034) (Additional file 1: Table S4).

Using *LEP* promoter methylation as a risk stratification, we reclassified eight IPSS-R-defined low-risk cases to a high-risk group and six IPSS-R-defined high-risk cases to a low-risk group (Additional file 1: Table S4).

Next, we combined CCUS and lower-risk MDS patients (*N* = 65) and found that a mean *LEP* promoter methylation below 39.57% at the time of diagnosis was able to stratify survival from death with a sensitivity of 63% and a specificity of 67% (positive predictive value = 71%, negative predictive value = 58%, AUC = 0.64, *p* = 0.021, Additional file 2: Fig. S4). Kaplan–Meier analysis indicated that patients with CCUS and lower-risk MDS with a *LEP* promoter methylation above the cutoff (39.57%) had inferior OS (*p* = 0.042, Additional file 2: Fig. S5). This high-risk subgroup included 16 CCUS patients and 15 lower-risk MDS patients (clinical characteristics of patients in the two methylation groups are shown in Additional file 1: Table S5). In this cohort, mean *LEP* promoter methylation below the cutoff was associated with having *DNMT3A* mutations (*p* = 0.013, Additional file 1: Table S5).

Interestingly, in a univariate Cox regression analysis both *LEP* promoter methylation and hemoglobin level were associated with OS. However, none were an independent marker in multivariate regression analysis (Additional file 1: Table S6). These results suggest that *LEP* promoter methylation may be associated with survival in CCUS and lower-risk MDS patients.

### Validation of the association of *LEP* promoter hypermethylation and poor prognosis in MDS

To further examine the finding that *LEP* promoter hypermethylation is associated with poor prognosis in MDS, we included data from an unpublished project where methylation in bone marrow MNCs was evaluated using EPIC BeadChip microarray in 55 MDS patients comprising the validation cohort (Fig. 1). We extracted data on methylation levels of four CpG sites covered by probes in the microarray. These four sites were located in the proximal promoter of *LEP* approximately 300 bp downstream of the eight CpG sites that were investigated in the training cohort by pyrosequencing (Additional file 2: Fig. S6).

**Fig. 6:**
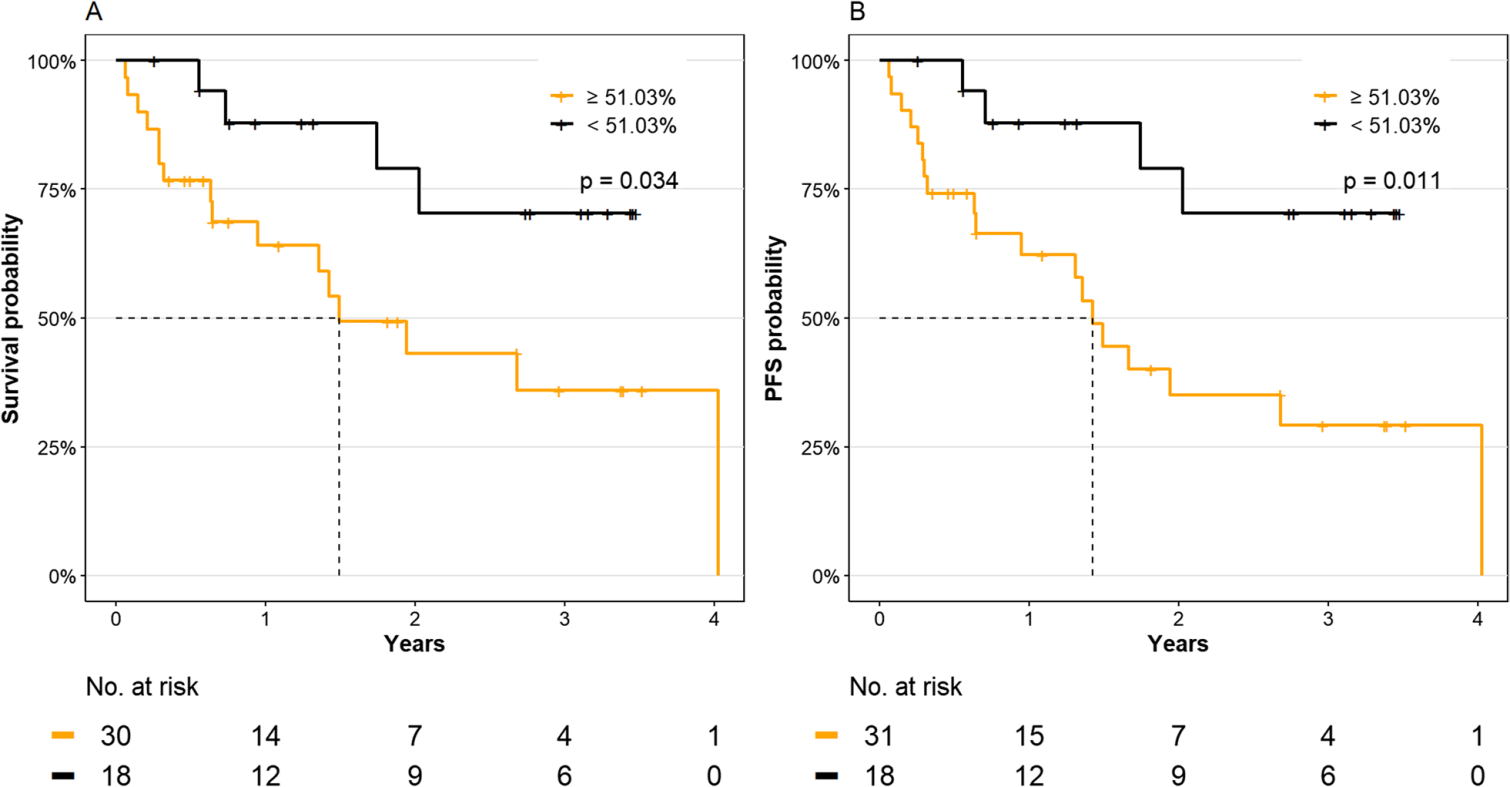
Impact of mean *LEP* promoter methylation above or below the cutoff of 51.03% in bone marrow MNCs from MDS patients (validation cohort) on **A** overall survival and **B** progression-free survival (PFS). MNC, mononuclear cells; MDS: myelodysplastic syndrome

We stratified the 55 MDS patients based on the methylation cutoff of 51.03% identified in the training cohort and evaluated their clinical course. It was confirmed in the validation cohort that MDS patients with a mean *LEP* promoter methylation above 51.03% had a significantly shorter OS and shorter PFS (*p* = 0.034 and *p* = 0.011, respectively, Fig. 6).

Forty patients (16 ICUS, 9 CCUS and 15 MDS patients, referred to as the comparison group in Fig. 1) had both peripheral blood and bone marrow samples collected, and there was a high correlation between the mean *LEP* promoter methylation levels measured using pyrosequencing in peripheral blood granulocytes and the mean *LEP* promoter methylation levels measured using the microarray in bone marrow MNCs (*R* = 0.71, *p* = 9.4 × 10^–7^, Additional file 2: Fig. S7). We observed slightly lower methylation levels in the peripheral blood granulocytes (median ± SD: 27% ± 20) compared to the bone marrow MNCs (median ± SD: 34% ± 17, paired Wilcoxon test *p* = 0.047).

### *LEP* promoter methylation increases during disease progression from MDS to secondary AML

**Fig. 7:**
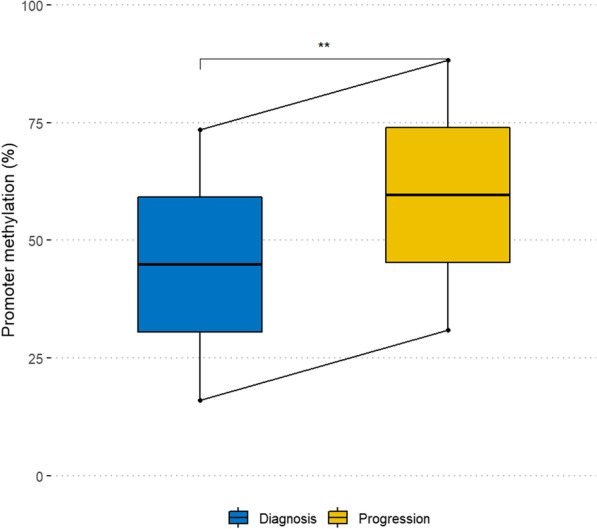
*LEP* promoter methylation at the time of diagnosis and the time of progression from MDS to secondary AML in four paired bone marrow samples. MDS: myelodysplastic syndrome; **indicate paired *t* test *p* ≤ 0.01

Sequential bone marrow samples from the time of diagnosis and time of progression were available from two MDS patients. When assessing *LEP* promoter methylation at the two time points using pyrosequencing, we found a significant increase in mean *LEP* promoter methylation at the time of progression from MDS to secondary AML (sAML) compared to the time of diagnosis with a relative increase in methylation of 20% in one patient and 92% in the other (Fig. 7).

### Plasma leptin levels are higher in patients with low *LEP* promoter methylation in their bone marrow

Next, we measured plasma leptin levels in peripheral blood from 28 ICUS, 21 CCUS and 13 MDS patients and three elderly healthy controls (Fig. 1).

**Fig. 8:**
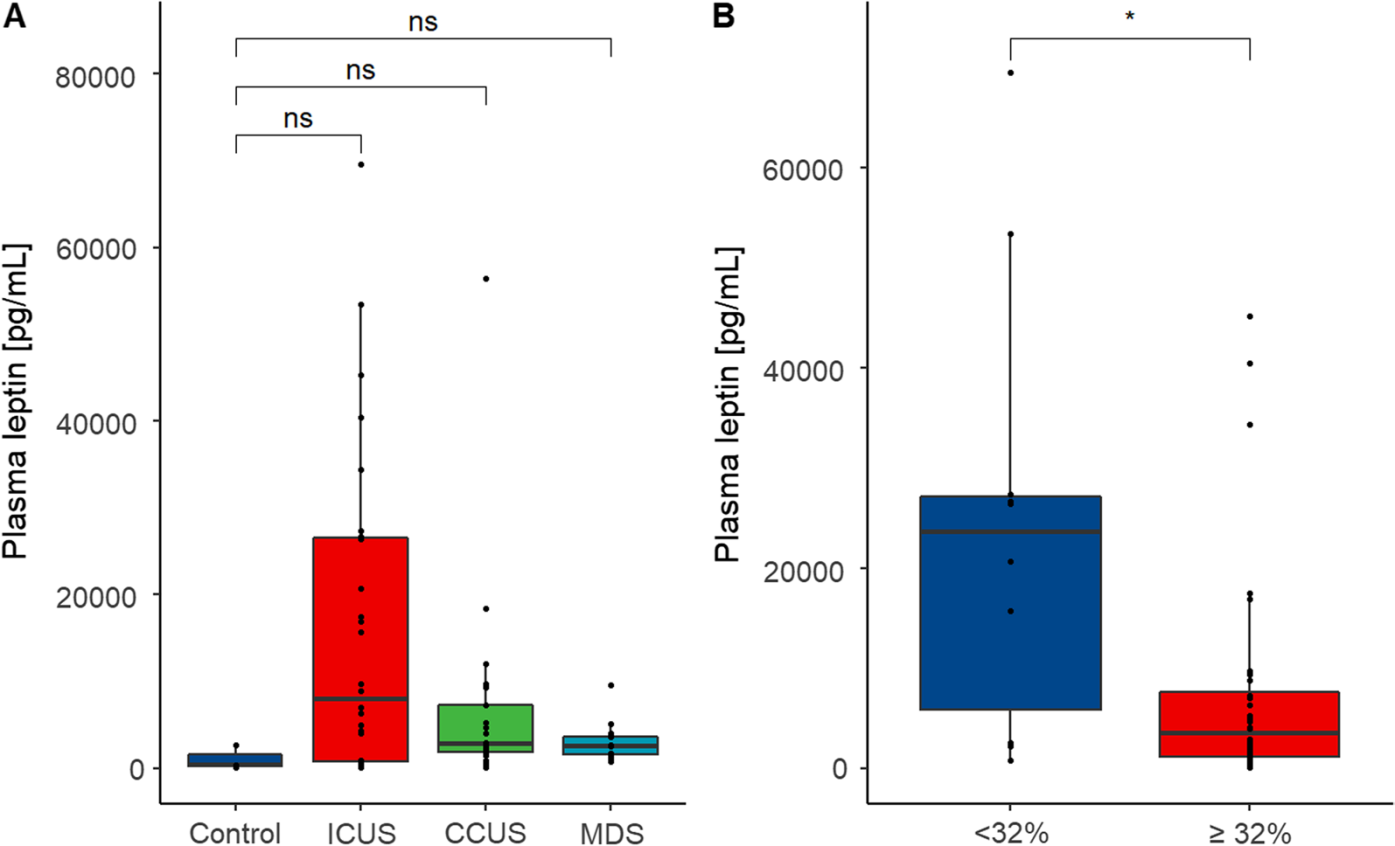
Plasma leptin levels at the time of diagnosis **A** in ICUS, CCUS and MDS patients and elderly healthy controls and **B** in patients with a mean *LEP* promoter methylation below or above 32% (1st quantile). ICUS: idiopathic cytopenia of undetermined significance; CCUS: clonal cytopenia of undetermined significance; MDS: myelodysplastic syndrome; *indicate *p* ≤ 0.05

Interestingly, ICUS patients had higher plasma leptin levels than CCUS and MDS patients, although this was not significant (Fig. 8A). Both methylation data and plasma leptin levels were available from 46 patients (24 ICUS, 14 CCUS, and eight MDS. Patients with a lower *LEP* promoter methylation in bone marrow MNCs (mean methylation below 32% corresponding to the 1st quantile among all patients) had a significantly higher plasma leptin level than patients with a *LEP* promoter methylation above 32% (*p* = 0.013, Fig. 8B).

Next, we investigated whether plasma leptin levels were related to systemic inflammation. We divided patients into a normal or inflammation group based on plasma levels of IFN-*γ*: patients were allocated to the inflammation group if the IFN-*γ* plasma level was above 3.9 pg/mL corresponding to the median + 2 SD of IFN-*γ* levels in the elderly healthy controls (2.3 pg/mL ± 1.6). We observed no differences in plasma leptin levels between the inflammatory groups (Additional file 2: Fig. S8). Furthermore, *LEP* promoter methylation in bone marrow MNCs was not correlated with IFN-*γ* plasma levels (Spearman correlation *r*_s_ =  − 0.07, *p* = 0.63).

Leptin levels were measured in both peripheral blood and bone marrow plasma from 8 of the patients and three elderly controls, and we found a highly significant correlation between leptin levels measured in the two compartments (*R* = 0.97, *p* = 7.4 × 10^–7^, Additional file 2: Fig. S9). A paired *t* test revealed that plasma levels in the two groups were not significantly different from each other.

## Discussion

With the widespread use of sequencing techniques, precursor states of myeloid neoplasms such as ICUS and CCUS are widely recognized. While these entities have similar clinical presentations, their malignant potential is different and the biological relationship between these disorders and MDS is not fully understood.

In this study, we measured DNA methylation of the *LEP* promoter in peripheral blood granulocytes from ICUS, CCUS and MDS patients to examine whether *LEP* promoter methylation may be an early event in leukemogenesis. We showed that patients with ICUS, CCUS and MDS all have a significantly hypermethylated *LEP* promoter compared to healthy controls. Furthermore, methylation levels seem to increase with disease severity as patients with MDS have a higher mean *LEP* promoter methylation than both CCUS and ICUS patients, and patients with CCUS have a higher mean *LEP* promoter methylation than patients with ICUS. This is well in line with the reported results of increased *LEP* promoter methylation in patients with AML [30] and MDS [29]. Together with previous data, our results suggest that *LEP* promoter hypermethylation is a frequent and early event in myeloid disorders.

The most common cytopenia in ICUS, CCUS and MDS is anemia which has been associated with a number of clinically relevant events in many epidemiological studies, most importantly increased risk of hospitalization and mortality [31]. Interestingly, we show that patients with anemia had a significantly hypermethylated *LEP* promoter compared to patients with a normal hemoglobin level.

Using follow-up data, we identified that a *LEP* promoter methylation cutoff of 51.03% in either peripheral blood granulocytes or bone marrow MNCs was able to stratify MDS patients with stable disease from those with progressive disease already at the time of diagnosis. MDS patients with a *LEP* promoter methylation above the cutoff had a higher risk of progression, and shorter OS and PFS. Furthermore, *LEP* promoter methylation was an independent risk factor for the progression of MDS in a multivariate Cox regression analysis. Using sequential samples, we show that *LEP* promoter methylation increased during the progression from MDS to sAML. However, this should be interpreted with precaution due to the small sample size (two patients).

A recently published study demonstrated that *LEP* promoter hypermethylation in bone marrow MNCs was associated with inferior OS and could serve as an independent prognostic predictor in AML [30]. However, the same authors reported that *LEP* hypermethylation in bone marrow MNCs from MDS patients was associated with a longer survival time in MDS but without being an independent prognostic marker [29]. Our study suggests a worse prognosis in MDS patients with a high *LEP* promoter methylation at the time of diagnosis. Some of these discrepancies may be due to differences in the clinical characteristics of the MDS patients and differences in the methods used to measure DNA methylation, and in the specific locus where methylation was investigated.

Bone marrow blast percentage is frequently used as an indication of disease progression. Interestingly, a high *LEP* promoter methylation in peripheral blood granulocytes was associated with a higher percentage of bone marrow blasts. In accordance with this, a previous study reported an association between *LEP* promoter hypermethylation and a higher percentage of bone marrow blasts in AML patients [30].

A limitation of our training cohort is the relatively small number of CCUS patients, the small number of progressive CCUS cases and the large variation in follow-up time. Since the diagnosis of CCUS and lower-risk MDS only differ by bone marrow morphology (i.e., below or above 10% dysplastic cells in the bone marrow, respectively), we decided to combine the cohorts of CCUS and lower-risk MDS patients which have been shown to have similar outcomes [32]. Kaplan–Meier analysis indicated that also CCUS and lower-risk MDS patients with a high *LEP* promoter methylation at the time of diagnosis had a shorter OS. These data further suggest that patients with a high *LEP* promoter methylation have a worse prognosis regardless of the malignant state.

*LEP* encodes the adipose tissue-derived hormone leptin. A recent study suggested that the epigenetic profile of *LEP* may be influenced by leptin serum levels in peripheral blood [33]. We observed a tendency toward higher plasma leptin levels in ICUS and CCUS patients than in elderly healthy controls although this was not significant. Several other studies report no difference in leptin levels in MDS [34-36] and AML [37] patients compared to healthy controls whereas two studies report lower leptin levels in AML [38, 39]. Interestingly, when we stratified patients based on their mean *LEP* promoter methylation in bone marrow MNCs, we found that patients with a low *LEP* promoter methylation had significantly higher plasma leptin levels compared to patients with a high *LEP* promoter methylation. This suggests that there may be an interplay between *LEP* promoter methylation in hematopoietic cells and circulating leptin levels. A previous study observed an inverse correlation between *LEP* promoter methylation and *LEP* expression in AML bone marrow MNCs [30].

Obesity confers an enhanced risk of multiple cancers including hematological malignancies [17-26]. It has been indicated that obesity-associated chronic inflammation procures a cancer-promoting state [40]. However, we did not observe any association between systemic inflammation (as defined by high IFN-*γ* plasma levels [41]), leptin plasma levels or *LEP* promoter methylation in bone marrow MNCs, respectively. The direct role of leptin in the initiation and progression of myeloid neoplasms is controversial and not fully understood. Several studies suggest a proliferative and/or anti-apoptotic effect of leptin on MDS [29] and AML [42-44] cells in vitro and one study suggests that leptin stimulates leukemic cell growth in vivo by stimulation of angiogenesis [45]. However, this is contradicted by the low or normal plasma leptin levels reported in several studies [34-39] and the *LEP* hypermethylation found in MDS patients in our study and reported by others [29]. Further studies are needed to elucidate the association between myeloid neoplasms, *LEP* methylation, and circulating leptin levels. In addition, future studies investigating whether the metabolic state (e.g., plasma glucose or insulin levels, etc.) influences *LEP* promoter methylation in blood cells of patients with ICUS, CCUS, or MDS could potentially reveal new mechanistic insight of the methylation changes. In summary, the data presented in this study indicate that hypermethylation of the *LEP* promoter is an early and frequent event in myeloid neoplasms and that it is associated with a worse prognosis in patients with MDS. This suggests that it may play a role in both initiation and progression of myeloid neoplasms. However, whether hypermethylation of the *LEP* promoter in blood and bone marrow cells is a pathogenic event itself or merely a consequence of the underlying disease is still unknown and an interesting target for future studies.

Furthermore, our study suggests that *LEP* promoter methylation may be a potential biomarker to identify patients at high risk of progression already at the time of diagnosis using only a peripheral blood sample. However, this needs confirmation in a larger cohort of patients.

## Conclusion

Our findings indicated that hypermethylation of the *LEP* promoter is an early and frequent event in myeloid neoplasms and was associated with a worse prognosis in MDS.

## Methods

### Study design

Study subjects were included from all hematological departments in Denmark and donated either peripheral blood, bone marrow aspirates or both at the time of diagnosis. Two patients with MDS also donated bone marrow aspirates at the time of progression. All participants provided written informed consent prior to participation and the study was conducted in accordance with Danish ethical regulation for work with human participants. The study was approved by the ethical committee with approval ID: CVK-1705391 and H-16022249. An overview of experiments, sample material and patient numbers are shown in Fig. 1.

The diagnoses of MDS patients were based on morphology and cytogenetics as in the WHO 2016 classification of MDS [3] and patients were risk-stratified with the revised international prognostic scoring system (IPSS-R) [46]. ICUS was defined as persistent cytopenia for more than six months with other common causes of cytopenia ruled out and with no known mutations in MDS-related genes in blood cells. CCUS was defined as persistent cytopenia for more than six months with other common causes of cytopenia ruled out and with the presence of somatic mutations in MDS-related genes in blood cells. Peripheral blood cytopenias were defined as platelet counts below 150 × 10^9^ cells/L, neutrophil counts below 1.8 × 10^9^ cells/L, and hemoglobin levels below 8.3 mmol/L or 7.3 mmol/L for men and women, respectively. Mutational status is reported for those patients in whom DNA from blood or bone marrow had been sequenced in the hematological departments.

### Sample material

*Training cohort* Peripheral blood was collected at the time of diagnosis from 65 patients with ICUS, 39 patients with CCUS and 57 patients with MDS as well as from 10 healthy controls. Granulocytes were isolated from peripheral blood by standard Ficoll-Paque PLUS (GE Healthcare) gradient separation followed by lysis of erythrocytes using red blood cell lysis buffer. Genomic DNA was extracted from the isolated granulocytes using AllPrep DNA/RNA/miRNA Universal Kit (Qiagen, Hilden, Germany) according to the manufacturer’s instructions.

*Validation cohort* From another unpublished project, we had methylation data available from bone marrow mononuclear cells (MNCs) of 55 MDS patients. Bone marrow aspirates were collected at the time of diagnosis and MNCs were isolated using standard Ficoll-Paque PLUS (GE Healthcare) gradient separation with subsequent T-cell depletion using RoboSep Human CD3 Positive Selection Kit II (StemCell Technologies, Vancouver, Canada) and RoboSep #20,000 (software version 4.6.0.1; StemCell Technologies) with a customized protocol using two-quadrant separation. DNA was extracted using QIAamp DNA Blood Mini Kit (Qiagen, Hilden, Germany). Methylation levels were measured as described in the “Data from Infinium MethylationEPIC array” section.

*Comparison group* 40 patients from the training cohort had DNA available from both T-cell-depleted bone marrow MNCs and peripheral blood granulocytes and were used to compare methylation levels across tissue and methods. DNA from bone marrow MNCs was isolated as described for the validation cohort.

*Sequential samples* Two MDS patients had a bone marrow sample collected both at the time of diagnosis and at the time of progression to sAML. DNA from bone marrow MNCs was isolated as described for the validation cohort.

*Plasma samples* Plasma from peripheral blood was collected at the time of diagnosis from 28 ICUS, 21 CCUS and 13 MDS patients and three elderly healthy controls. In addition, bone marrow plasma was also collected from eight of the patients with CCUS and MDS and from the three elderly healthy controls.

### Pyrosequencing of the *LEP* promoter

DNA methylation of eight CpG sites located 324–349 bp upstream of the transcription start site within a 262 bp region of the *LEP* promoter was investigated in peripheral blood granulocytes from the training cohort and in the sequential bone marrow MNC samples using a pyrosequencing assay designed with PyroMark Assay design software (Qiagen, primers are listed in Additional file 1: Table S6). Genomic DNA (250–300 ng) was bisulfite-converted using the EZ DNA Methylation-Lightning™ Kit (Zymo Research Irvine, California, USA). Bisulfite-converted DNA was amplified with primer sets and the PyroMark PCR kit according to the manufacturer’s instructions. Pyrosequencing was performed using the PyroMark Q24 instrument and PyroMark Gold Q24 reagents. Runs were quality controlled using the PyroMark Q24 software (version 2.0.7; Qiagen, Hilden, Germany). The mean methylation level of the eight CpG sites was calculated for each sample and used for further analysis.

### Data from infinium MethylationEPIC array

Methylation levels were measured in DNA from bone marrow MNCs using the Illumina HumanMethylationEPIC BeadChip (Illumina San Diego, California, USA) according to the manufacturer’s protocol.

Data were quality controlled using the minfi R package v.1.36.0 [6]. Data were normalized using functional normalization [7, 8]. Probes with detection *p* values > 0.01, bead count < 3 in at least 5% of samples, non-CpG sites, probes targeting sex chromosomes, SNPs < 5 bp from the target CpG [9] and probes that previously showed binding to multiple target CpGs [10] were excluded using the ChAMP R package v. 2.20.1. Beta-values of four CpG sites located − 61 to − 32 bp upstream of the transcription start site of the *LEP* promoter were extracted for each sample and mean methylation of these four sites in each sample were used for further analysis.

### Plasma leptin and IFN-γ

Leptin levels were measured in plasma from peripheral blood and bone marrow using a U-plex chemiluminescence-based assay (catalog # K151ACL-1) from Meso Scale Discovery (MSD, Gaithersburg, MD, USA). The leptin detection range was 11.9–48,800 pg/mL.

Interferon-*γ* (IFN-*γ*) levels were measured in plasma from peripheral blood using a V-plex chemiluminescence-based assay (catalog # K151A9H-1) from Meso Scale Discovery (MSD). The IFN-*γ* detection range was 0.369–1510 pg/mL.

All samples were analyzed in duplicates. Analyses were done using a QuickPlex SQ 120 instrument (MSD) and the DISCOVERY WORKBENCH® 4.0 software.

### Statistical analyses

Statistical analyses were performed using R version 4.1.0.

Wilcoxon rank-sum and chi-square tests were used for the comparison of nonparametric continuous variables and categorical variables, respectively, unless otherwise stated. The receiver operating characteristic (ROC) curve was used to determine an appropriate cutoff for mean *LEP* promoter methylation level at the time of diagnosis to predict disease outcome using the cutpointr R-package v.1.1.2. The optimal cutoff was determined using the Youden index. Follow-up data were included for patients where information about progression and/or survival was available. Cumulative incidence curves were used to estimate the cumulative risk of progression, and a comparison was made with Gray’s test. Death of any cause was considered a competing risk in the analysis. Time to progression was calculated as the time of diagnosis to the time of progression. Progression was defined as CCUS patients getting a later diagnosis of either MDS or AML and MDS patients with blast transformation or with progression to AML. The outcome was censored if a patient had not progressed by the time of the last follow-up, if a patient was lost to follow-up or if a patient had a hematopoietic stem cell transplantation (HSCT). Survival analysis of the effect of *LEP* promoter methylation on OS and PFS was done by Kaplan–Meier and Cox regression analyses (univariate and multivariate). For OS, the outcome was censored if a patient was alive by the time of the last follow-up, if a patient was lost to follow-up or if a patient had a HSCT. For PFS, the outcome was censored if a patient was alive and had not progressed by the time of the last follow-up, if a patient was lost to follow-up or if a patient had a HSCT. “IPSS-R low” was defined as IPSS-R score ≤ 3 unless otherwise stated. A *p* value ≤ 0.05 was considered significant.

## Supplementary Information

**Additional file 1: Table S1.** List of reported mutations in CCUS and MDS patients from training. **Table S2.** Multivariate regression analysis of LEP promoter methylation. **Table S3. **Univariate Regression Analyses of Disease Outcome in MDS patients from the training cohort. **Table S4.** Clinical characteristics of MDS patients separated based on methylation group. **Table S5.** Clinical characteristics of CCUS and lower-risk MDS patients separated based on methylation group. **Table S6.** Cox Regression Analysis of overall survival in CCUS and lower-risk. **Table S7.** List of pyrosequencing primers.

**Additional file 2: Fig. S1.** LEP promoter methylation in the training cohort in **A** patients with thrombocytopenia or normal platelet counts and **B** patients with neutropenia or normal neutrophil counts. **Fig. S2.** Disease outcome in the training cohort. **A** Overall survival and **B** cumulative incidence of progression in ICUS, CCUS and MDS. ICUS: idiopathic cytopenia of undetermined significance; CCUS: clonal cytopenia of undetermined significance; MDS: myelodysplastic syndrome. **Fig. S3.** LEP promoter methylation at the time of diagnosis in CCUS patients who during follow-up remain stable or progress. CCUS: clonal cytopenia of undetermined significance. **Fig. S4. **ROC curve analysis of LEP promoter methylation separating survival and death during follow-up of CCUS and lower-risk MDS in the training cohort. ROC, receiver operating characteristic curve; AUC, Area under the curve. **Fig. S5. **Overall survival in patients with CCUS or lower-risk MDS from the training cohort stratified based on their mean LEP promoter methylation at the time of diagnosis with 39.57% as cutoff. **Fig. S6. **Schematization of **A** the LEP promoter and **B** the regions we investigated using, respectively, pyrosequencing spanning from -349 to -324 and EPIC-microarray spanning from -61 to -32 in respect to transcription start site. **Fig. S7**. Correlation between mean LEP promoter methylation measured in eight CpG sites in peripheral blood granulocytes using pyrosequencing and four CpG sites measured in bone marrow mononuclear cells using an EPIC-microarray. **Fig. S8**. Plasma leptin levels in patients with systemic inflammation compared to patients with normal IFN-γ levels. IFN-γ: Interferon-γ. **Fig. S9**. Correlation between log-transformed peripheral blood plasma leptin and bone marrow plasma leptin levels in paired samples. Pearson’s correlation coefficient R=1.

## Data Availability

The raw and processed data generated in this study as well as other individual-level data are only available under restricted access since these data are considered sensitive personal data according to Danish Law and the European Union General Data Protection Regulation (GDPR) and thus cannot be shared with third parties without prior approval. To access data, an application must be sent to Kirsten.Groenbaek@regionh.dk. Access can only be granted for research purposes, and only if a data processor or data transfer agreement can be made in accordance with Danish and European law at the given time. The expected timeframe from response until access is granted is ~ 6 months.
