# Supplementary material for: LEP promoter methylation in the initiation and progression of clonal cytopenia of undetermined significance and myelodysplastic syndrome
Source: Clin Epigenetics. 2023 May 26;15:91. doi: 10.1186/s13148-023-01505-w (PMC10224308; doi:10.1186/s13148-023-01505-w)
Supplement: Supplementary file 1 — Additional file 1: Table S1. List of reported mutations in CCUS and MDS patients from training. Table S2. Multivariate regression analysis of LEP promoter methylation. Table S3. Univariate Regression Analyses of Disease Outcome in MDS patients from the training cohort. Table S4. Clinical characteristics of MDS patients separated based on methylation group. Table S5. Clinical characteristics of CCUS and lower-risk MDS patients separated based on methylation group. Table S6. Cox Regression Analysis of overall survival in CCUS and lower-risk. Table S7. List of pyrosequencing primers. [file 13148_2023_1505_MOESM1_ESM.pdf]

**Supplementary Table 1. List of reported mutations in CCUS and MDS patients from training cohort**			
**Characteristic**	**CCUS, N = 39**[1]	**MDS, N = 57**[1]	**p-value**[2]
***TET2* mutations**			0.407
No	23 (59%)	14 (70%)	
Yes	16 (41%)	6 (30%)	
Missing	0	37	
***DNMT3A* mutations**			0.076
No	27 (69%)	18 (90%)	
Yes	12 (31%)	2 (10%)	
Missing	0	37	
***IDH1* mutations**			0.544
No	37 (95%)	20 (100%)	
Yes	2 (5.1%)	0 (0%)	
Missing	0	37	
***IDH2* mutations**			0.263
No	38 (97%)	18 (90%)	
Yes	1 (2.6%)	2 (10%)	
Missing	0	37	
***SF3B1* mutations**			0.166
No	37 (95%)	16 (80%)	
Yes	2 (5.1%)	4 (20%)	
Missing	0	37	
***SRSF2* mutations**			0.563
No	30 (77%)	14 (70%)	
Yes	9 (23%)	6 (30%)	
Missing	0	37	
***RUNX1* mutations**			0.325
No	37 (95%)	17 (85%)	
Yes	2 (5.1%)	3 (15%)	
Missing	0	37	
***ASXL1* mutations**			0.483
No	33 (85%)	15 (75%)	
Yes	6 (15%)	5 (25%)	
Missing	0	37	
***JAK2* mutations**			
No	39 (100%)	20 (100%)	
Missing	0	37	
***TP53* mutations**			>0.999
No	37 (95%)	19 (95%)	
Yes	2 (5.1%)	1 (5.0%)	
Missing	0	37	
***U2AF1* mutations**			0.263
No	38 (97%)	18 (90%)	

Yes	1 (2.6%)	2 (10%)	
Missing	0	37	
***ETV6* mutations**			0.339
No	39 (100%)	19 (95%)	
Yes	0 (0%)	1 (5.0%)	
Missing	0	37	
***ZRSR2* mutations**			>0.999
No	36 (92%)	18 (90%)	
Yes	3 (7.7%)	2 (10%)	
Missing	0	37	
***EZH2* mutations**			>0.999
No	38 (97%)	20 (100%)	
Yes	1 (2.6%)	0 (0%)	
Missing	0	37	
***CBL* mutations**			0.544
No	37 (95%)	20 (100%)	
Yes	2 (5.1%)	0 (0%)	
Missing	0	37	
***CEBPa1* mutations**			
No	39 (100%)	20 (100%)	
Missing	0	37	
***SETBP* mutations**			>0.999
No	38 (97%)	20 (100%)	
Yes	1 (2.6%)	0 (0%)	
Missing	0	37	
***NRAS* mutations**			
No	39 (100%)	20 (100%)	
Missing	0	37	
***KRAS* mutations**			
No	39 (100%)	20 (100%)	
Missing	0	37	
***GATA2* mutations**			0.339
No	39 (100%)	19 (95%)	
Yes	0 (0%)	1 (5.0%)	
Missing	0	37	
***SH2B3* mutations**	1 (6.7%)	0 (0%)	0.469
Missing	24	40	

[1] n (%)

[2] Pearson's Chi-squared test; Fisher's exact test

CCUS: clonal cytopenia of undetermined significance, MDS: myelodysplastic syndrome.

Supplementary Table 2. Multivariate regression analysis of *LEP* promoter methylation

Characteristic	Whole cohort			Patients		
	**Estimate**	**95% CI**	**p-value**	**Estimate**	**95% CI**	**p-value**
Diagnosis						
CTRLS	—	—				
ICUS	12	5.2, 18	**<0.001**	25	3.7, 46	**0.022**
CCUS	20	14, 26	**<0.001**	36	16, 57	**<0.001**
MDS	-5.2	-16, 5.2	0.326	48	27, 68	**<0.001**
Age	0.09	-0.08, 0.26	0.315	0.07	-0.13, 0.27	0.504
Sex						
Female	—	—		—	—	
Male	-1.7	-6.8, 3.4	0.503	-2	-7.7, 3.7	0.496
Hgb				0.08	-1.8, 1.9	0.928
ANC				0.8	-0.64, 2.2	0.272
Platelets				-0.02	-0.04, 0.01	0.151

CI: confidence interval; Hgb: hemoglobin; ANC: absolute neutrophil count; CTRLS: healthy controls; ICUS: idiopathic cytopenia of undetermined significance; CCUS: clonal cytopenia of undetermined significance; MDS: myelodysplastic syndrome.

Supplementary table 3. Univariate Regression Analyses of Disease Outcome in MDS patients from the training cohort

Characteristic	Time to Death				Time to Progression			
	**N**	**HR**	**95% CI**	**p-value**	**N**	**HR**	**95% CI**	**p-value**
LEP promoter methylation	51				47			
Below cut-off		—	—			—	—	
Above cut-off		2.96	1.38, 6.36	**0.005**		4.16	1.61, 10.8	**0.003**
Age	53	1	0.97, 1.04	0.755	49	1.01	0.97, 1.05	0.703
Sex	53				49			
Female		—	—			—	—	
Male		0.84	0.39, 1.81	0.659		1.56	0.52, 4.68	0.427
Hgb	48	0.68	0.51, 0.90	**0.007**	45	1.19	0.90, 1.56	0.223
ANC	46	1.1	0.92, 1.32	0.305	43	1.18	0.95, 1.46	0.131
Platelets	49	1	0.99, 1.00	0.168	46	1	0.99, 1.00	0.336
BM blast %	49	1.07	1.00, 1.16	0.058	45	1.19	1.09, 1.30	**<0.001**
IPSS-R	40				44			
Low		—	—			—	—	
Intermediate		2.25	0.91, 5.56	0.08		3.98	1.41, 11.2	**0.009**
High		2.45	0.85, 7.02	0.095		3.39	1.09, 10.5	**0.034**

HR:Hazard Ratio; CI: Confidence Interval; below cut-off: mean LEP promoter methylation < 51.03%; Above cut-off: mean LEP promoter methylation ≥ 51.03%; Hgb: hemoglobin; ANC: absolute neutrophil count; IPSS-R: Revised International Prognostic Scoring System BM: bone marrow; MDS: myelodysplastic syndrome.

Supplementary table 4. Clinical characteristics of MDS patients separated based on methylation group

**Characteristic**	**Below cut-off, N = 34[1]**	**Above cut-off, N = 21[1]**	**p-value[2]**
**Age**	73.5 (26.0, 96.0)	74.0 (43.0, 90.0)	0.538
**Sex**			0.428
Female	8	7	
Male	26	14	
**Hgb**	6.2 (4.9, 14.0)	6.2 (4.8, 11.1)	0.517
Missing	7	2	
**ANC**	1.4 (0.5, 5.4)	1.5 (0.3, 11.4)	0.963
Missing	8	2	
**Platelets**	105.0 (6.0, 666.0)	120.0 (19.0, 379.0)	0.706
Missing	7	1	
**BM blast %**	3.0 (0.0, 15.0)	5.0 (0.0, 18.0)	**0.034**
Missing	5	3	
**IPSS-R Category**			0.612
Low	16	8	
Intermediate	5	5	
High	6	5	
Missing	7	3	
**No. of mutations**			**0.024**
0	3	0	
1	3	2	
2	3	1	
3	0	5	
4	1	0	
Missing	24	13	
***TET2* mutations**			>0.999
0	7	6	
1	3	2	
Missing	24	13	
***DNMT3A* mutations**			>0.999
0	9	7	
1	1	1	
Missing	24	13	
***IDH1* mutations**			>0.999
0	10	8	
1	0	0	
Missing	24	13	
***IDH2* mutations**			0.183
0	10	6	
1	0	2	
Missing	24	13	
***U2AF1* mutations**			>0.999
0	10	8	
1	0	0	
Missing	24	13	
***SF3B1* mutations**			0.588
0	7	7	
1	3	1	
Missing	24	13	

***ASXL1* mutations**			0.608
0	8	5	
1	2	3	
Missing	24	13	
***TP53* mutations**			>0.999
0	9	8	
1	1	0	
Missing	24	13	
***RUNX1* mutations**			0.069
0	10	5	
1	0	3	
Missing	24	13	

[1] Median (Range); n

[2] Wilcoxon rank sum test; Pearson's Chi-squared test; Fisher's exact test

Hgb: hemoglobin, ANC: absolute neutrophil count, BM: bone marrow, below cut-off: mean *LEP* promoter methylation < 51.03%, Above cut-off: mean *LEP* promoter methylation ≥ 51.03%.

Supplementary table 5. Clinical characteristics of CCUS and lower-risk MDS patients separated based on methylation group

Characteristic	Below cut-off, N = 31[1]	Above cut-off, N = 31[1]	p-value[2]
**Diagnosis**			0.118
CCUS	22	16	
MDS	9	15	
**Age**	70.0 (27.0, 81.0)	73.0 (51.0, 89.0)	0.239
**Sex**			0.783
Female	10	9	
Male	21	22	
**Hgb**	6.3 (4.5, 8.7)	6.4 (4.7, 8.6)	0.602
**ANC**	2.1 (0.9, 5.8)	1.7 (0.6, 8.0)	0.159
**Platelets**	135.0 (6.0, 666.0)	107.0 (39.0, 427.0)	0.36
**BM blast %**	1.0 (0.0, 9.0)	0.5 (0.0, 10.0)	0.955
Missing	14	15	
**No. of mutations**			**0.004**
0	2	3	
1	16	6	
2	9	9	
3	0	6	
4	0	3	
Missing	4	4	
***TET2* mutations**	7	12	0.154
Missing	4	4	
***DNMT3A* mutations**	11	3	**0.013**
Missing	4	4	
***IDH1* mutations**	2	0	0.491
Missing	4	4	
***IDH2* mutations**	0	3	0.236
Missing	4	4	
***U2AF1* mutations**	1	0	>0.999
Missing	4	4	
***SF3B1* mutations**	2	4	0.669
Missing	4	4	
***ASXL1* mutations**	3	8	0.091
Missing	4	4	
***TP53* mutations**	3	0	0.236
Missing	4	4	
***RUNX1* mutations**	0	4	0.111
Missing	4	4	

[1] n; Median (Range)

[2] Pearson's Chi-squared test; Wilcoxon rank sum test; Fisher's exact test

CCUS: clonal cytopenia of undetermined significance, MDS: myelodysplastic syndrome, Hgb: hemoglobin, ANC: absolute neutrophil count, BM: bone marrow, below cut-off: mean LEP promoter methylation < 39.5%, Above cut-off: mean LEP promoter methylation ≥ 39.5%.

Supplementary Table 6. Cox Regression Analysis of overall survival in CCUS and lower-risk MDS

Characteristic	Univariate				Multivariate		
	**N**	**HR**	**95% CI**	**p-value**	**HR**	**95% CI**	**p-value**
LEP promoter methylation	62						
Below cut-off		—	—		—	—	
Above cut-off		2.07	1.01, 4.22	**0.047**	3.54	0.69, 18.2	0.131
Age	65	1.03	0.99, 1.07	0.2	1.03	0.95, 1.12	0.49
Sex	65						
Female		—	—		—	—	
Male		1.05	0.51, 2.13	0.9	0.33	0.09, 1.24	0.101
Hgb	65	0.56	0.40, 0.77	**<0.001**	0.66	0.34, 1.29	0.224
ANC	64	0.98	0.81, 1.19	0.9	1.28	0.84, 1.94	0.254
Platelets	65	1	0.99, 1.00	0.2	1	0.99, 1.00	0.505
BM blast %	36	1.13	0.87, 1.46	0.4	1.18	0.85, 1.65	0.329

HR: Hazard Ratio; CI: Confidence Interval; Hgb: hemoglobin; ANC: absolute neutrophil count; IPSS-R: revised international prognostic scoring system; BM: bone marrow; CCUS: clonal cytopenia of undetermined significance; MDS: myelodysplastic syndrome.

Supplementary Table 7. List of pyrosequencing primers.

Primer	Id	Sequence
PCR	F1	AGAAGGGGTGGGATTTTAGAA
PCR	R1	ACCAACCCCTTAAAAAAATACTT
Sequencing	S1	ACCCCTTAAAAAAATACTTC
