# Supplementary material for: LEP promoter methylation in the initiation and progression of clonal cytopenia of undetermined significance and myelodysplastic syndrome
Source: Clin Epigenetics. 2023 May 26;15:91. doi: 10.1186/s13148-023-01505-w (PMC10224308; doi:10.1186/s13148-023-01505-w)
Supplement: Supplementary file 2 — Additional file 2: Fig. S1. LEP promoter methylation in the training cohort in A patients with thrombocytopenia or normal platelet counts and B patients with neutropenia or normal neutrophil counts. Fig. S2. Disease outcome in the training cohort. A Overall survival and B cumulative incidence of progression in ICUS, CCUS and MDS. ICUS: idiopathic cytopenia of undetermined significance; CCUS: clonal cytopenia of undetermined significance; MDS: myelodysplastic syndrome. Fig. S3. LEP promoter methylation at the time of diagnosis in CCUS patients who during follow-up remain stable or progress. CCUS: clonal cytopenia of undetermined significance. Fig. S4. ROC curve analysis of LEP promoter methylation separating survival and death during follow-up of CCUS and lower-risk MDS in the training cohort. ROC, receiver operating characteristic curve; AUC, Area under the curve. Fig. S5. Overall survival in patients with CCUS or lower-risk MDS from the training cohort stratified based on their mean LEP promoter methylation at the time of diagnosis with 39.57% as cutoff. Fig. S6. Schematization of A the LEP promoter and B the regions we investigated using, respectively, pyrosequencing spanning from -349 to -324 and EPIC-microarray spanning from -61 to -32 in respect to transcription start site. Fig. S7. Correlation between mean LEP promoter methylation measured in eight CpG sites in peripheral blood granulocytes using pyrosequencing and four CpG sites measured in bone marrow mononuclear cells using an EPIC-microarray. Fig. S8. Plasma leptin levels in patients with systemic inflammation compared to patients with normal IFN-γ levels. IFN-γ: Interferon-γ. Fig. S9. Correlation between log-transformed peripheral blood plasma leptin and bone marrow plasma leptin levels in paired samples. Pearson’s correlation coefficient R=1. [file 13148_2023_1505_MOESM2_ESM.pdf]

LEP **promoter methylation in the initiation and progression of clonal cytopenia of undetermined significance and myelodysplastic syndrome**

Supplementary figures

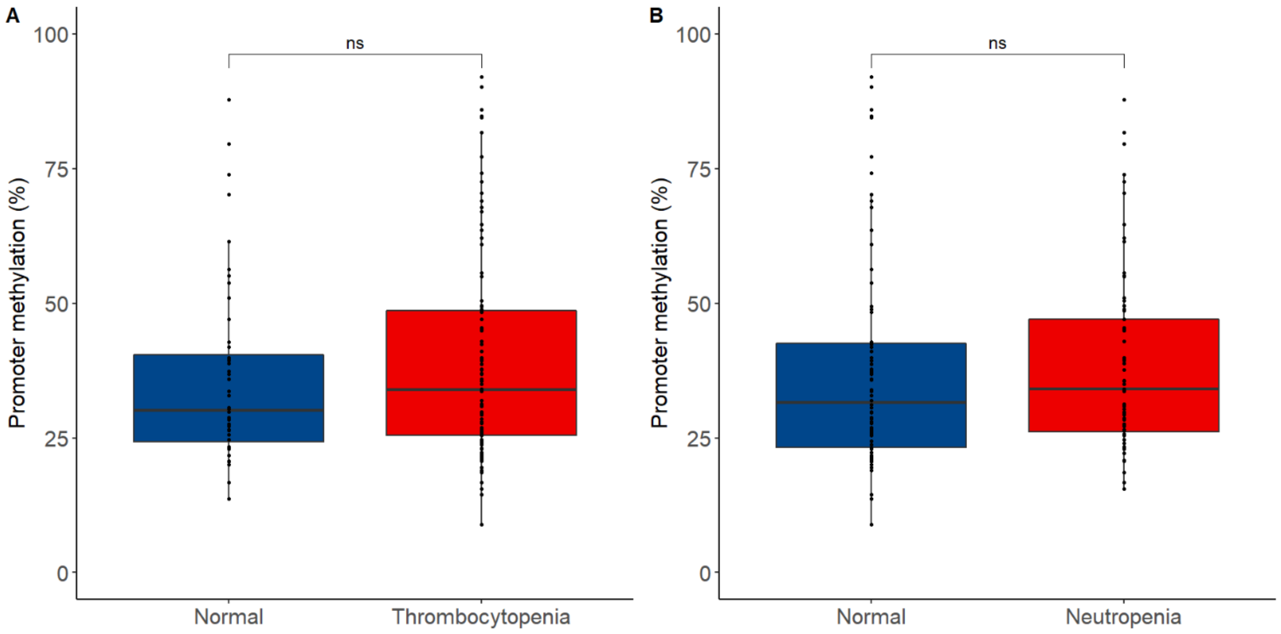

Supplementary figure 1. *LEP* promoter methylation in the training cohort in A) patients with thrombocytopenia or normal platelet counts and B) patients with neutropenia or normal neutrophil counts.

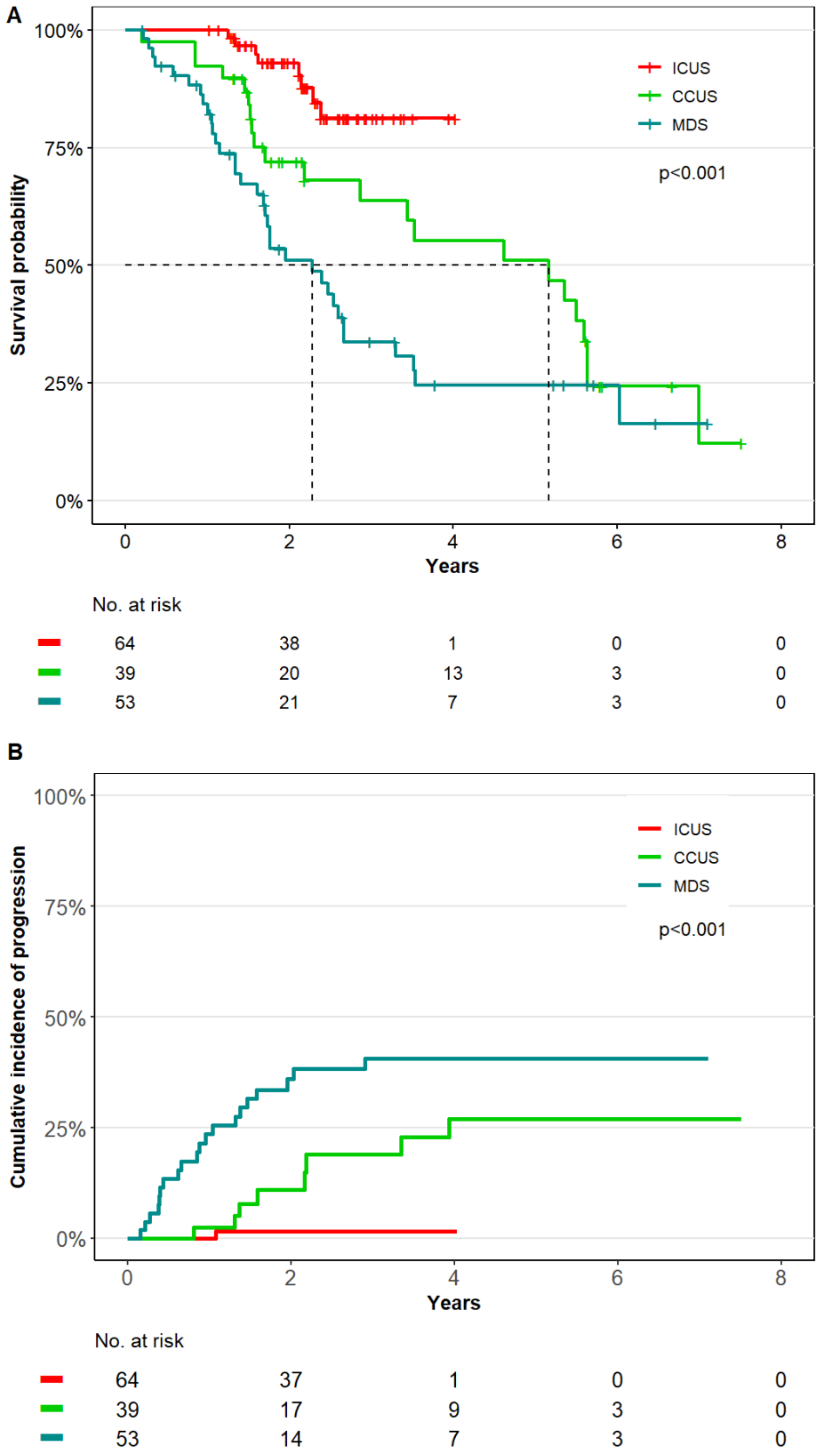

Supplementary figure 2. Disease outcome in the training cohort. **A)** Overall survival and **B)** cumulative incidence of progression in ICUS, CCUS and MDS. ICUS: idiopathic cytopenia of undetermined significance; CCUS: clonal cytopenia of undetermined significance; MDS: myelodysplastic syndrome.

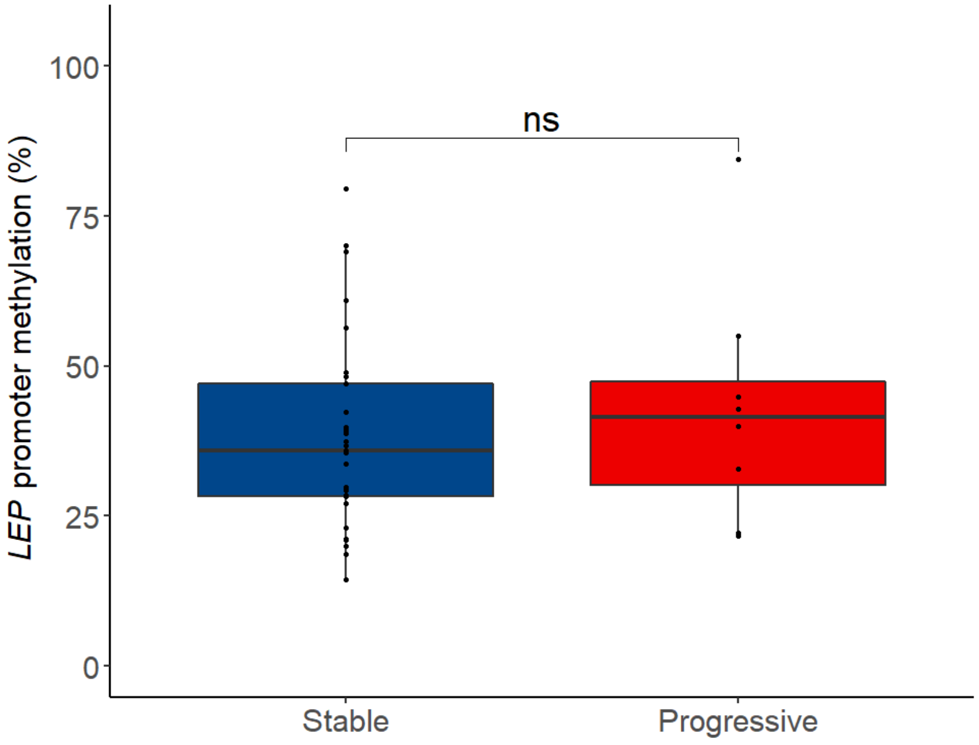

Supplementary figure 3. *LEP* promoter methylation at time of diagnosis in CCUS patients who during follow-up remain stable or progress. CCUS: clonal cytopenia of undetermined significance.

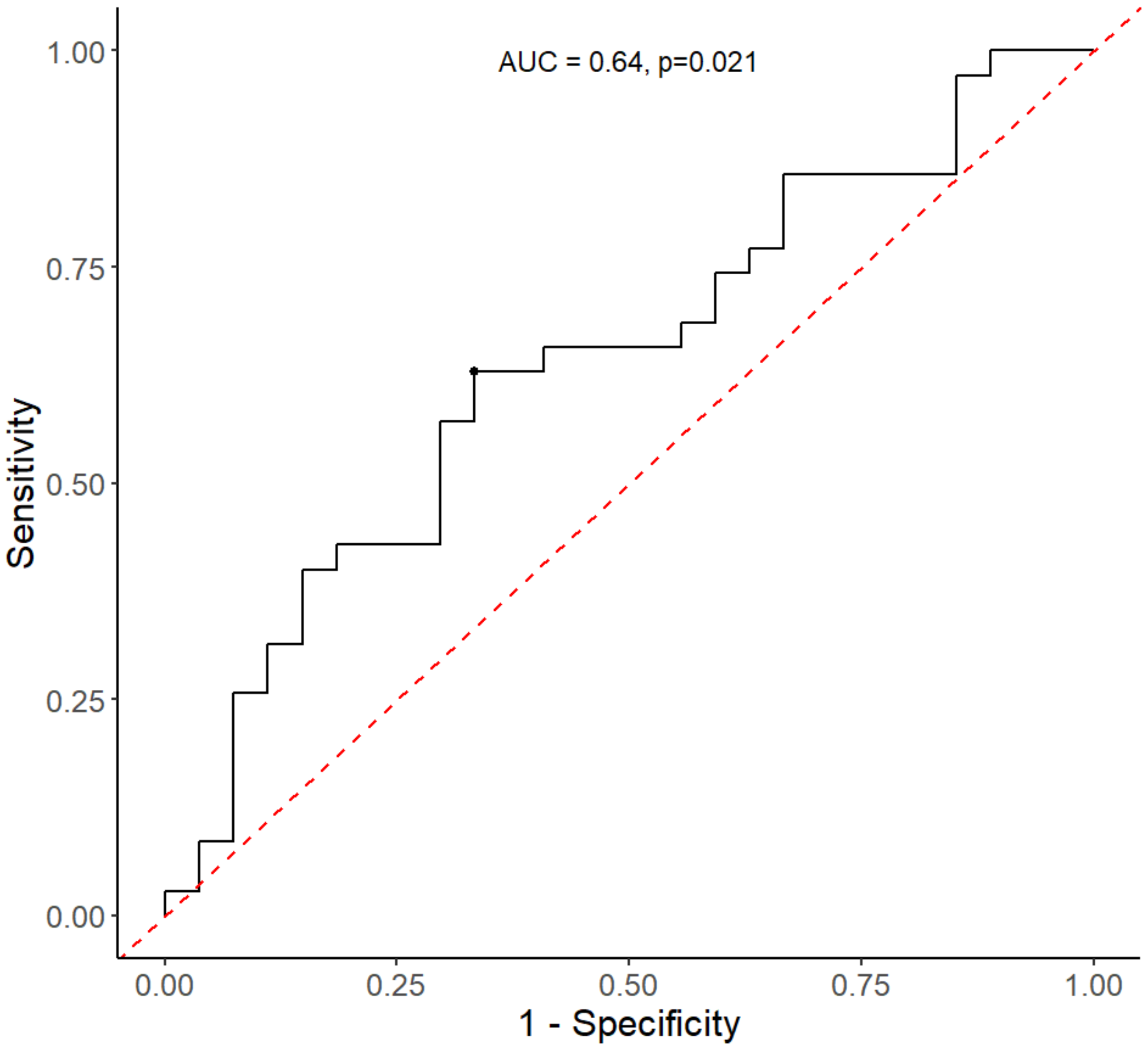

Supplementary figure 4. ROC curve analysis of *LEP* promoter methylation separating survival and death during follow-up of CCUS and lower-risk MDS in the training cohort. ROC, receiver operating characteristic curve; AUC, Area under the curve.

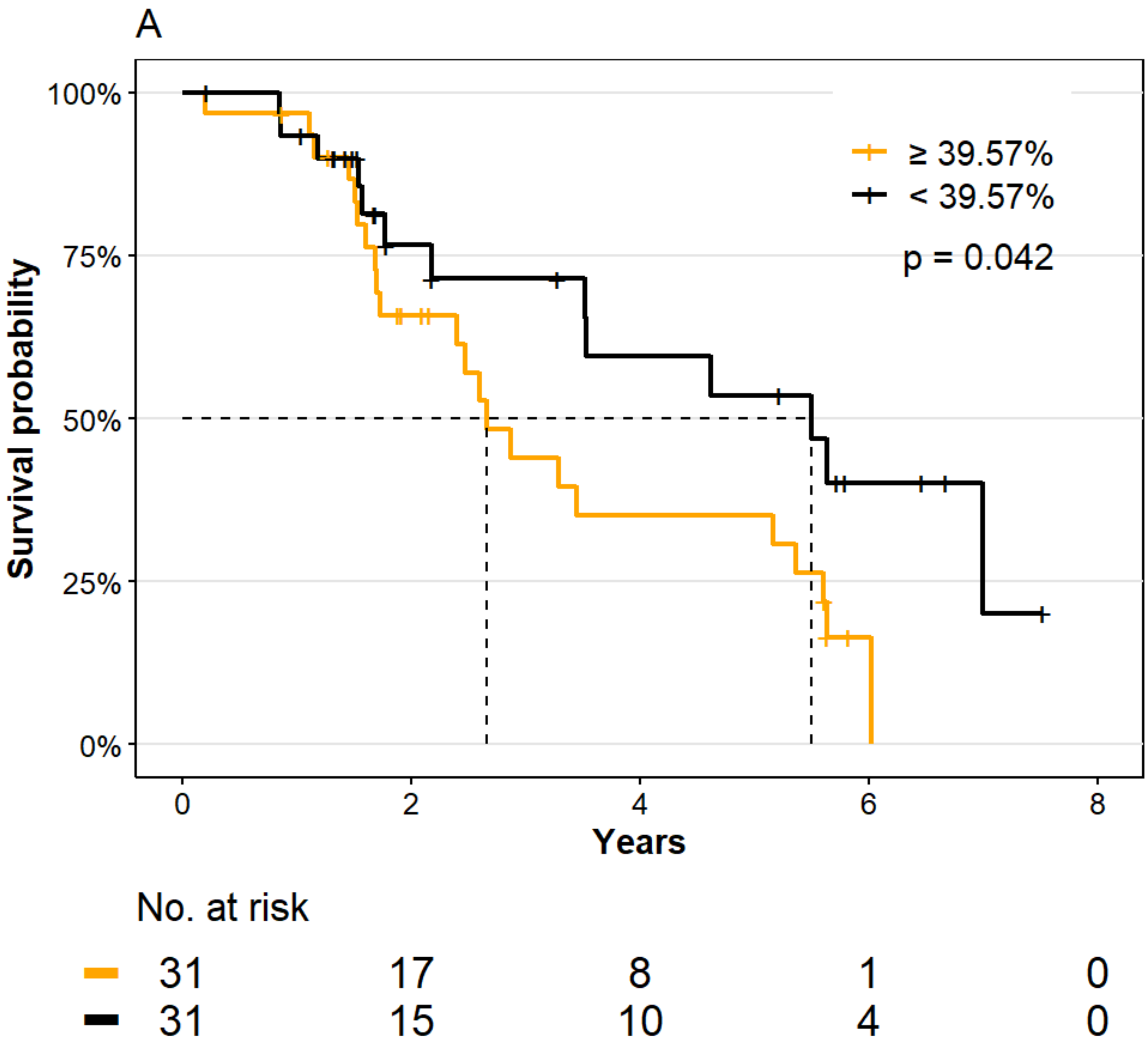

Supplementary figure 5. Overall survival in patients with CCUS or lower-risk MDS from the training cohort stratified based on their mean *LEP* promoter methylation at time of diagnosis with 39.57% as cut-off.

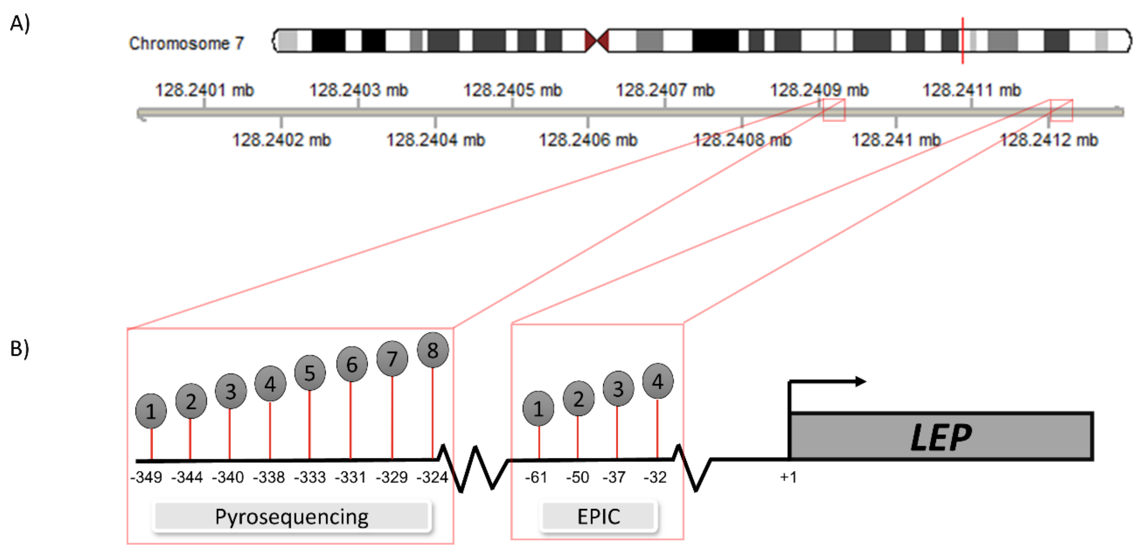

Supplementary figure 6. Schematization of A) the *LEP* promoter and B) the regions we investigated using, respectively, pyrosequencing spanning from -349 to -324 and EPIC-microarray spanning from -61 to -32 in respect to transcription start site (+1).

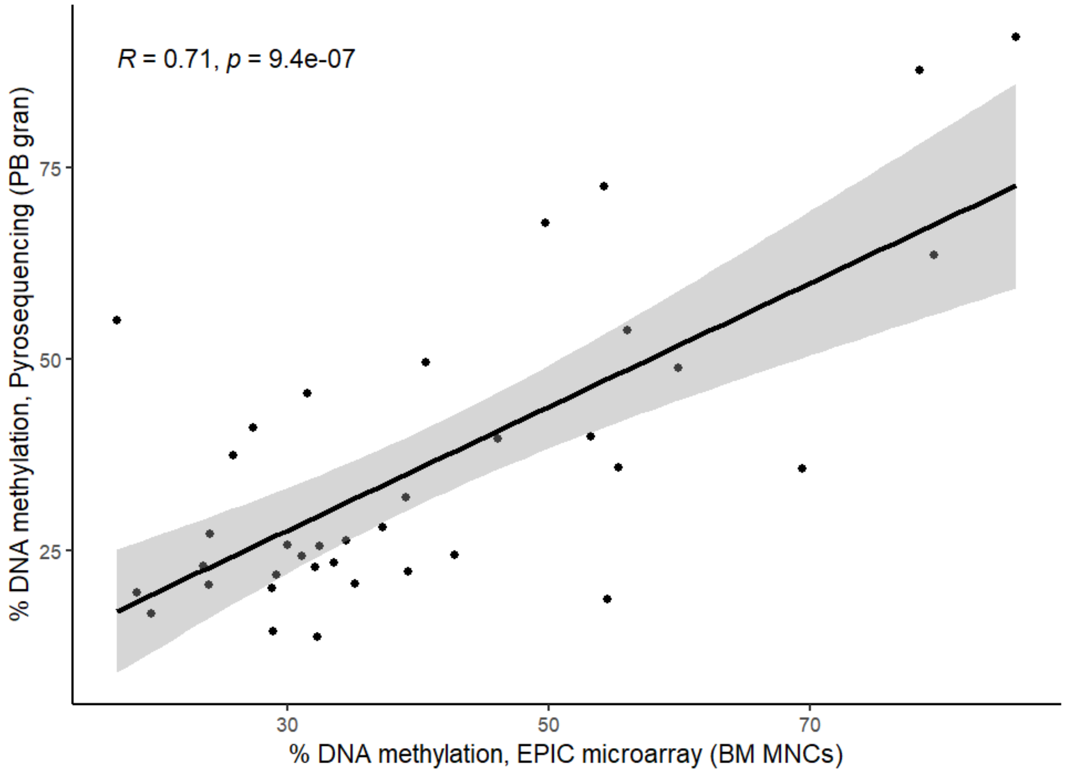

Supplementary figure 7. Correlation between mean *LEP* promoter methylation measured in eight CpG sites in peripheral blood granulocytes using pyrosequencing and four CpG sites measured in bone marrow mononuclear cells using an EPIC-microarray.

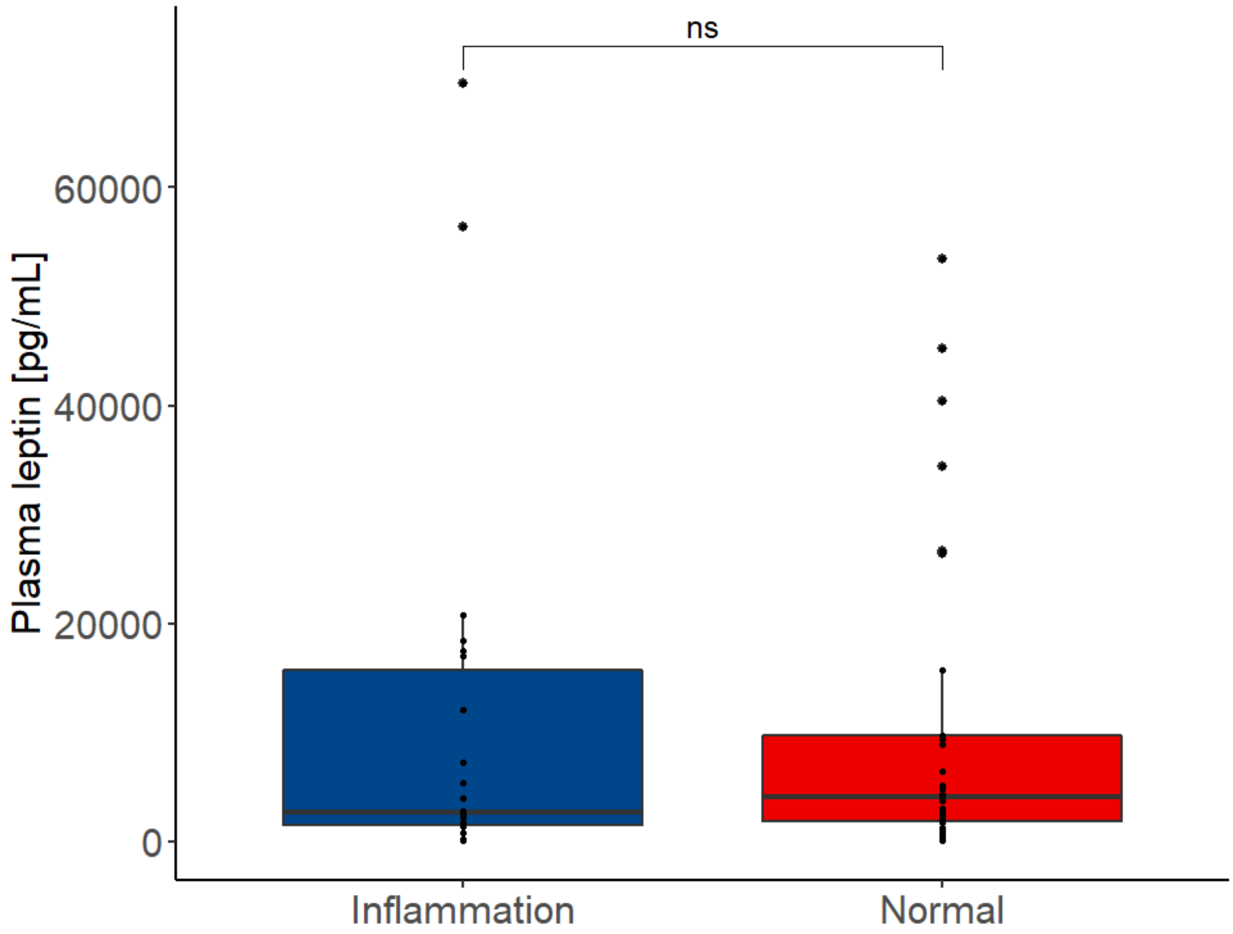

Supplementary figure 8. Plasma leptin levels in patients with systemic inflammation (plasma IFN-γ > 3.9 pg/mL) compared to patients with normal IFN-γ levels. IFN-γ: Interferon-γ.

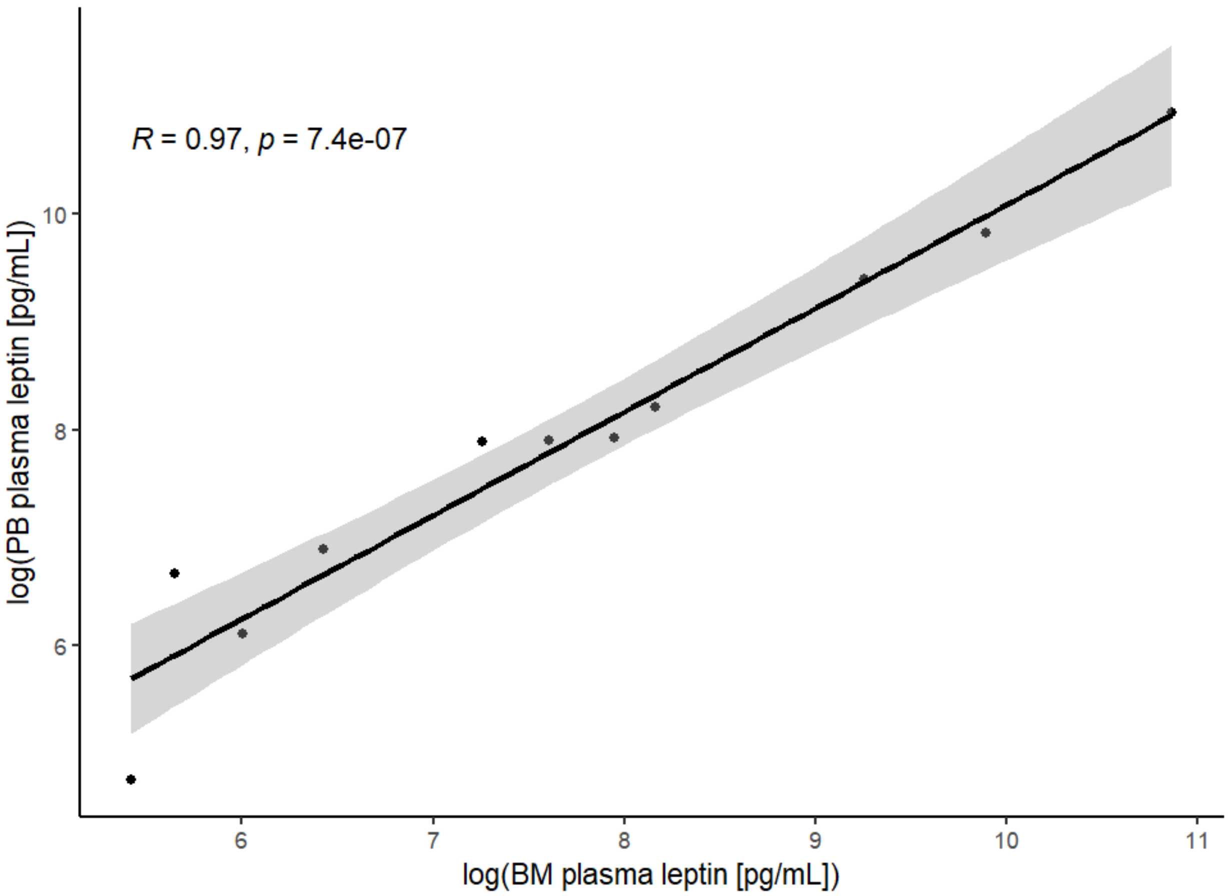

Supplementary figure 9. Correlation between log-transformed peripheral blood (PB) plasma leptin and bone marrow (BM) plasma leptin levels in paired samples.
